# Supplementary material for: The Construction and Exploration of a Comprehensive MicroRNA Centered Regulatory Network in Foxtail Millet (Setaria italica L.)
Source: Front Plant Sci. 2022 May 6;13:848474. doi: 10.3389/fpls.2022.848474 (PMC9121102; doi:10.3389/fpls.2022.848474)
Supplement: Supplementary file 1 [file Data_Sheet_1.zip › Sit-miR2118a_Seita.3G275400.1_956_TPlot.pdf]

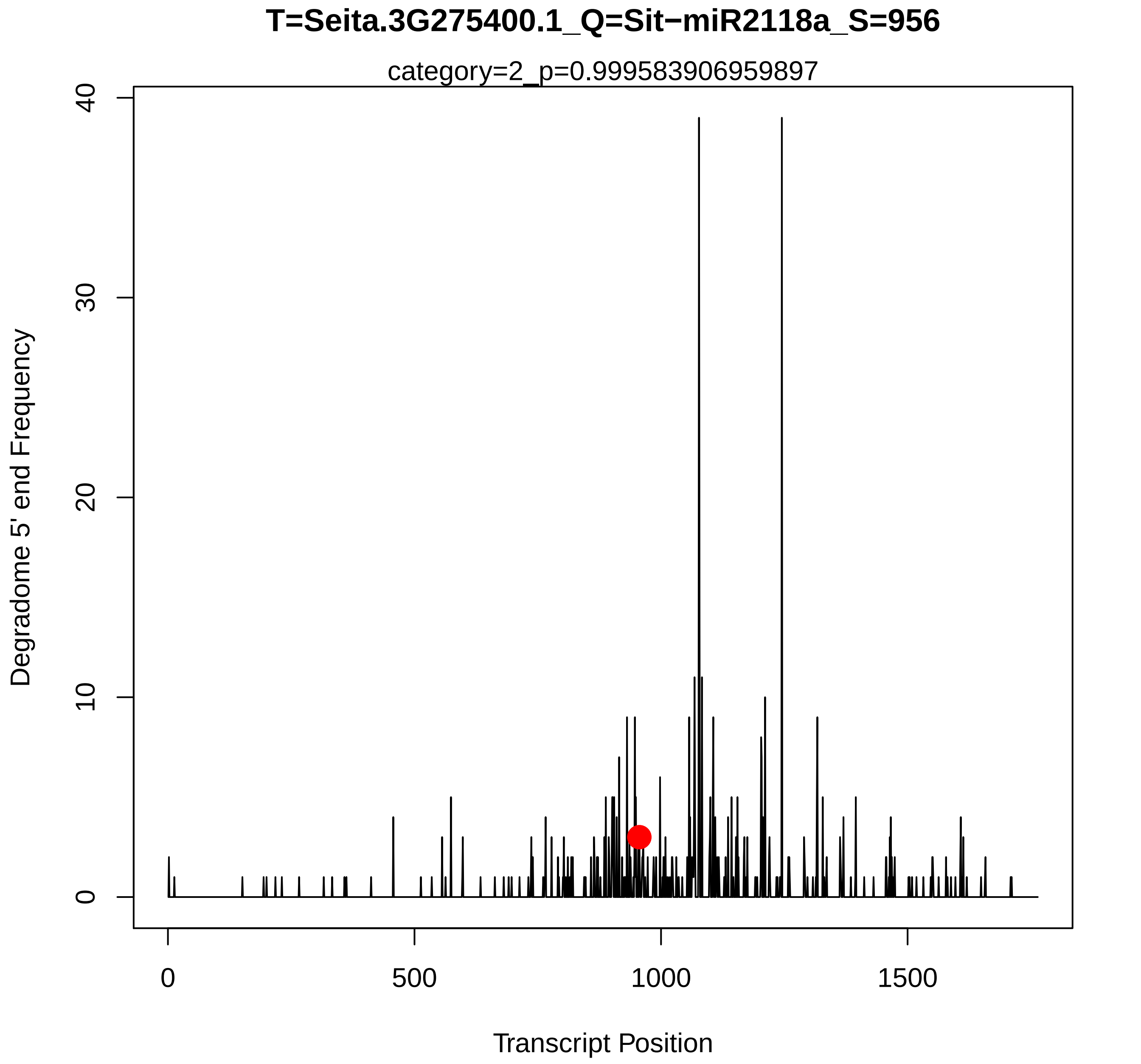

T=Seita.3G275400.1_Q=Sit−miR2118a_S=956
category=2_p=0.999583906959897
Degradome 5' end Frequency
0
10
20
30
40
0
500
1000
1500
Transcript Position
